# Dynamic mode coupling in terahertz metamaterials

**DOI:** 10.1038/srep10823

**Published:** 2015-06-02

**Authors:** Xiaoqiang Su, Chunmei Ouyang, Ningning Xu, Siyu Tan, Jianqiang Gu, Zhen Tian, Ranjan Singh, Shuang Zhang, Fengping Yan, Jiaguang Han, Weili Zhang

**Affiliations:** 1 Center for Terahertz Waves and College of Precision Instrument and Optoelectronics Engineering, Tianjin University, and Key Laboratory of Optoelectronics Information and Technology, Ministry of Education of China, Tianjin 300072, People’s Republic of China; 2 School of Electrical and Computer Engineering, Oklahoma State University, Stillwater, Oklahoma 74078, USA; 3 Key Lab of All Optical Network and Advanced Telecommunication Network of EMC, Institute of Lightwave Technology, Beijing Jiaotong University, Beijing 100044, People’s Republic of China; 4 Center for Disruptive Photonic Technologies, Division of Physics and Applied Physics, School of Physical and Mathematical Sciences, Nanyang Technological University, 21 Nanyang Link, Singapore 637371; 5 School of Physics and Astronomy, University of Birmingham, Birmingham B15 2TT, UK

## Abstract

The near and far field coupling behavior in plasmonic and metamaterial systems have been extensively studied over last few years. However, most of the coupling mechanisms reported in the past have been passive in nature which actually fail to control the coupling mechanism dynamically in the plasmonic metamaterial lattice array. Here, we demonstrate a dynamic mode coupling between resonators in a hybrid metal-semiconductor metamaterial comprised of metallic concentric rings that are physically connected with silicon bridges. The dielectric function of silicon can be instantaneously modified by photodoped carriers thus tailoring the coupling characteristics between the metallic resonators. Based on the experimental results, a theoretical model is developed, which shows that the optical responses depend on mode coupling that originates from the variation of the damping rate and coupling coefficient of the resonance modes. This particular scheme enables an in-depth understanding of the fundamental coupling mechanism and, therefore, the dynamic coupling enables functionalities and applications for designing on-demand reconfigurable metamaterial and plasmonic devices.

## Introduction

In recent years, the concept of artificially engineered materials, known as metamaterials, has provided access to an even broader range of novel functionalities, such as negative refractive index^1^,^2^,^3^, perfect focusing^4^, cloaking^5^,^6^,^7^, absorbers^8^, sensing^9^,^10^, analogues of electromagnetically induced transparency^11^,^12^,^13^,^14^,^15^,^16^,^17^, and phase discontinuities^18^,^19^,^20^,^21^. The unique properties offered by metamaterials depend not only on the electromagnetic response of the individual meta-atoms, for instance the shape, the size, the conducting properties of the meta-atoms, and the surrounding environment^22^,^23^, but also on the coupling mechanism between the meta-atoms that form the two or three dimensional lattice array. Since the size of the meta-atoms is much smaller than the wavelength of light, interactions of the near-field distribution of the neighboring meta-atoms become very critical. As a result, the resonance properties of metamaterials can be substantially manipulated compared to those of an individual meta-atom. Thus, the near field interactions between adjacent meta-atoms have received considerable attention. Aside from the interesting fundamental phenomena that arise in these coupled meta-atoms, an understanding of the coupling mechanisms is believed to provide essential insight into novel design and applications of metamaterials based functional devices. Much of the published work has explored interactions between different resonators, where the coupling were studied passively by varying the geometrical parameters^24^,^25^,^26^,^27^.

In this article, we present a novel class of infrared light-driven active terahertz metamaterials that enable an observation of a dynamic coupling behavior between different resonators. The proposed planar metamaterial unit cell is composed of three concentric metallic square rings bridged by semiconductor inclusions. Since the adjacent metallic rings are bridged with the photoactive silicon (Si) and one can mold the photo-induced carrier density by varying the optical illumination, the coupling between the square rings would thus be tailored with photoexcitation. The advantage of such design is that the coupling can be actively controlled and reconfigured. This work provides a profound understanding of the fundamental coupling mechanisms in metamaterials and would certainly benefit the active and passive device designs with desirable optical properties.

### Design and experimental results

**Figure 1:**
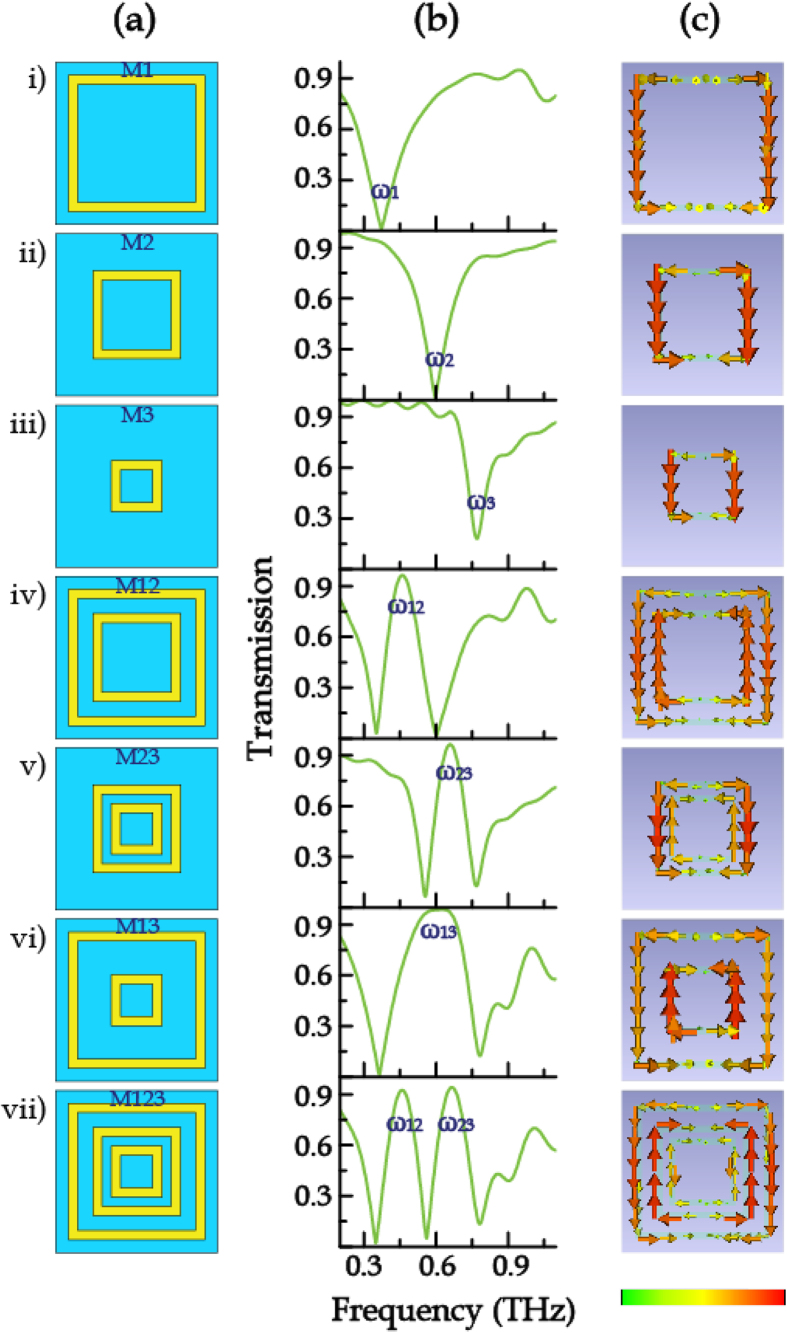
(**a**) Schematic diagram of the mode-coupling metamaterial design. (**b**) Numerical simulation results (**c**) Corresponding surface current distributions.

As shown in Fig. 1(a i–iii), three individual square closed ring resonators (CRRs), termed as M1, M2 and M3 with side-length of 104, 72, and 52 μm, respectively, are initially investigated. Numerical simulations of spectral responses of these samples were performed using commercial software CST Microwave Studio. The planar metallic CRRs were made of Aluminum with a conductivity of 3.72 × 10^7^ S m^−1^ and printed on the sapphire substrate (*ε*_sap_ = 9.48) with a square lattice period of *P* = 125 μm. We calculated the transmission spectra of M1, M2 and M3 when illuminated by a plane wave with the wave vector oriented parallel to the *z* axis and the electric field oriented parallel to the *x* axis. Figure 1(b i–iii) shows that the sharp transmission dip exists at *ω*_1_ = 0.357 THz, *ω*_2_ = 0.593 THz, and *ω*_3_ = 0.771 THz for M1, M2 and M3, respectively. It is found that the resonance frequency shows a notable blue shift when the CRRs are downsized while the other parameters remain unchanged.

In order to study the coupling behaviors between different resonators, combined configurations between two of M1, M2 and M3 are investigated. When M1 and M2 are placed together to form a concentric dual-ring resonator M12, as shown in Figs. 1(a iv) and (b iv), a distinct transparency window appeared at *ω*_12_ = 0.461 THz between *ω*_1_ and *ω*_2_. The coupling between M1 and M2 also gives rise to a slight red shift of *ω*_1_ and a slight blue shift of *ω*_2_. Figure 1(c iv) shows the calculated electric field distributions at *ω*_12_ and it is evident that anti-parallel currents are produced in M1 and M2. Since the outer ring experiences a stronger coupling to the incident terahertz wave, the inner ring, which is only weakly coupled to the incident light through ring to ring capacitive interaction, generates currents opposite to those in the outer ring through near-field coupling. The destructive interference of the scattered fields between the two resonators leads to a pronounced transparency window^28^,^29^. Attributed to the coupling effect, the response of M12 is not a simple summation of that of M1 and M2.

The similar phenomena were also observed in M23 (combination of M2 and M3, *ω*_23_ = 0.659 THz) and M13 (combination of M1 and M3, *ω*_13_ = 0.631 THz), as shown in Fig. 1(v, vi). Furthermore, when all the three square rings were combined together, named as M123, the transmission spectrum exhibited two transparency windows resulting from the interplay of the coupling between two sets of adjacent rings M1-M2 and M2-M3, as shown in Fig. 1(vii). In this case, as M1-M3 undergoes much weaker coupling to the radiation field compared to M1-M2 and M2-M3, hence the transmission response for this metamaterial consisted of multiple concentric rings mainly derives from the mode coupling of adjacent resonators.

**Figure 2:**
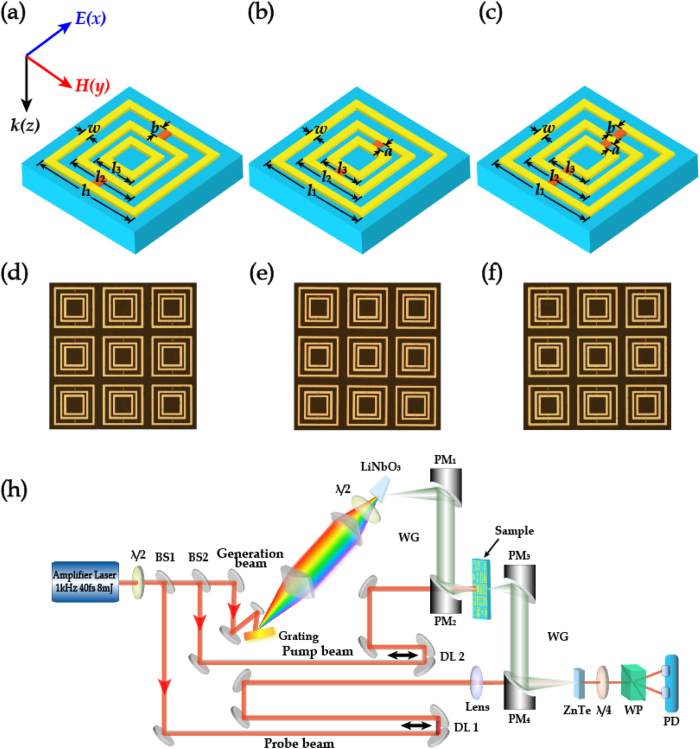
(a–c) Schematic diagram of M123-12, M123-23 and M123-123 metamaterial unit cells, respectively. The geometrical parameters are: *P*_x_  = *P*_y_ = 125 μm, *l*_1_ = 104 μm, *l*_2_ = 72 μm, *l*_3_ = 52 μm, *w* = 5 μm, *a* = 15 μm, *b* = 27 μm. (**d**–**f**) Microscopic images of the active mode-coupling metamaterials. (**h**) Experimental diagram of the optical-pump terahertz-probe (OPTP) measurement.

In order to gain an insight into a dynamic coupling effect, we further proposed a metamaterial structure fabricated on a Si-on-sapphire (SOS) wafer, which is comprised of 500-nm-thick undoped Si film and 495-μm-thick sapphire substrate. By implanting photoactive Si islands that connect the two adjacent concentric SRRs in the structure of M123, three types of active metamaterials with varying positions of the Si islands are investigated. The low order resonance M12, high order resonance M23 or both can be selectively manipulated, respectively. Figures 2(a), (b) and (c) show the proposed structures M123-12, M123-23 and M123-123, respectively. The fabrication of all the metamaterial samples began with patterning of Si islands by reactive ion etching, followed by deposition of 200-nm-thick Al to form the square rings using photolithography. The optical microscope images of these metamaterial samples are shown in Figs. 2(d) to (f).

An optical-pump terahertz-probe (OPTP) system, as illustrated in Fig. 2(g), was used to carry out the measurements with the polarization of the incident terahertz electric field parallel to the photosensitive Si islands. In the measurements, a laser beam coming from a Ti: sapphire regenerative amplifier (Coherent Lasers) with a pulse duration of 40 fs at 800 nm and a repetition rate of 1 kHz was split into three beams for terahertz generation, detection and metamaterial photodoping, respectively. The generated terahertz radiation was collimated and focused by two off-axis parabolic mirrors. The spot size of the focused terahertz beam on the metamaterial samples was 1.8 mm in diameter, while the optical pump beam exciting the samples had a spot diameter of 10 mm, ensuring uniform pump-terahertz illumination. Importantly, the pump pulse must reach the metamaterial sample ahead of the terahertz pulse within the carrier life time of Si which is about 1 ms to enable the Si islands to stay at the excited state. A bare sapphire wafer identical to the sample substrate served as a reference. The transmission spectrum was extracted from Fourier transforms of the measured time-domain electric fields, which was defined as 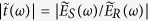, where 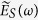 and 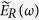 were the Fourier transformed electric fields through the sample and reference, respectively.

**Figure 3:**
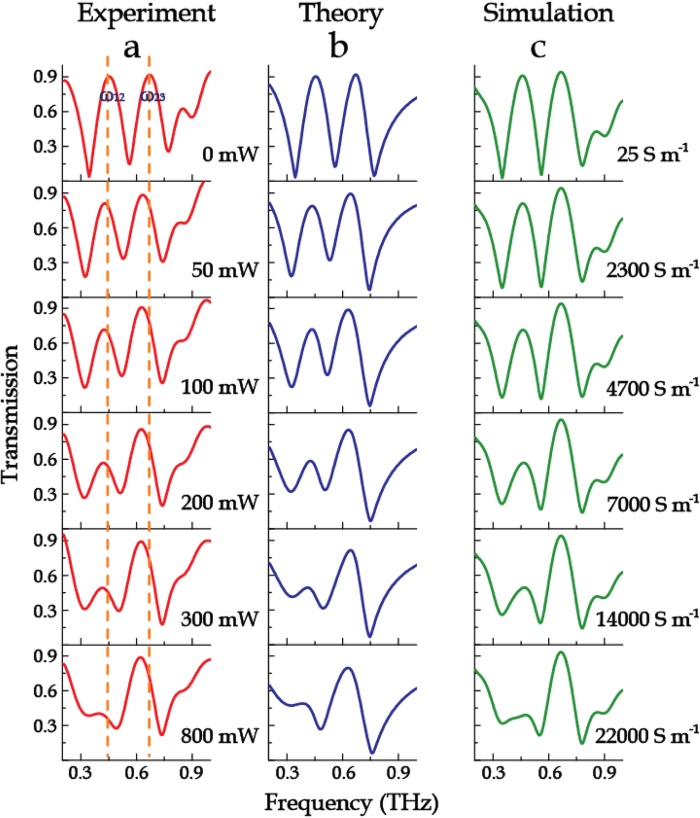
Evolution of transmission spectra of sample M123-12. (**a**) Normalized transmission spectra as a function of photoexcitation power. (**b**) Corresponding theoretical fitting results with the coupled Lorentz model. (**c**) Numerical simulations with various conductivities of the Si islands.

**Figure 4:**
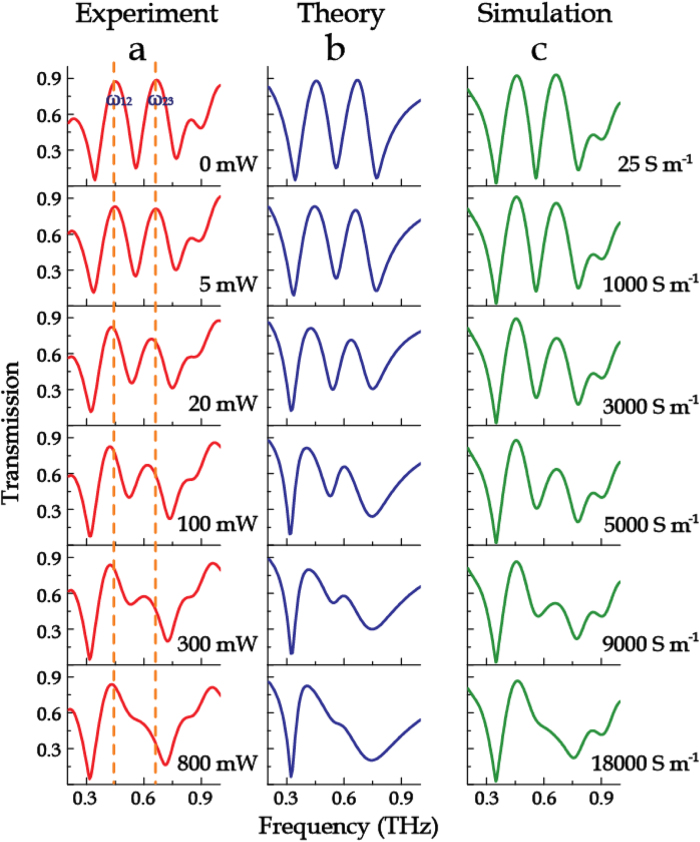
Evolution of transmission spectra of sample M123-23. (**a**) Normalized transmission spectra as a function of photoexcitation power. (**b**) Corresponding theoretical fitting results with the coupled Lorentz model. (**c**) Numerical simulations with various conductivities of the Si islands.

**Figure 5:**
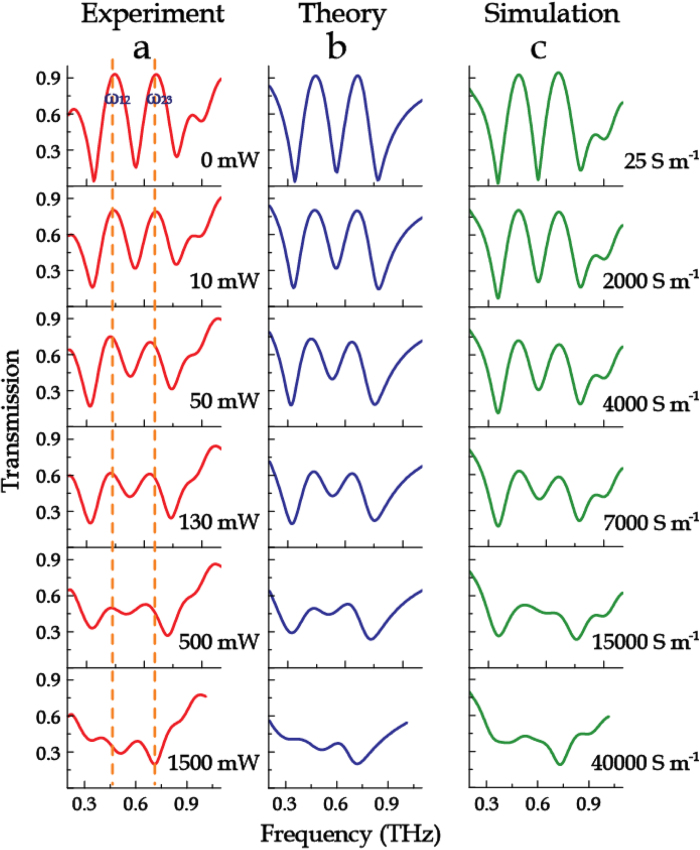
Evolution of transmission spectra of sample M123-123. (**a**) Normalized transmission spectra as a function of photoexcitation power. (**b**) Corresponding theoretical fitting results with the coupled Lorentz model. (**c**) Numerical simulations with various conductivities of the Si islands.

By varying the optical pump power, dynamic modulations of the transmission properties of the metamaterial samples were observed, as shown in Figs. 3, 4, 5. Without photo irradiation, the pronounced characteristic peaks ω_12_, ω_23_ and resonance dips ω_1_, ω_2_, ω_3_ are identical with the structure shown in Fig. 1(vii) in which no photoactive Si islands are employed. For the sample M123-12 with the Si islands connecting the outermost two rings, as the optical pump power was increased from 0 to 800 mW, the low frequency transmission window decreased gradually until it disappeared (Fig. 3(a)). Additionally, the transmission spectra of M123-23 with Si islands bridging the innermost two rings were also measured under photoexciation. With increasing pump power, the high frequency transparency window was dynamically controlled, as illustrated in Fig. 4(a). Interestingly, both the cases possess a remarkable feature that has no impact on adjacent transparency window during the amplitude modulation. When the Si islands connected all the three rings (sample M123-123), the two transmission windows were significantly reduced in magnitude under optical excitation (see Fig. 5(a)). At the maximum excitation of 1500 mW, the two transmission windows nearly disappeared.

### Theoretical calculations and discussion

To clarify the underlying physical mechanism of the experimental phenomena, the coupled Lorentz model is adopted to illustrate the mode resonances and their coupling characteristics with various optical excitation fluences. The resonances and their mutual coupling behavior of the metamaterial samples are analytically described by the following model:


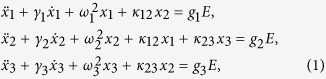


where *x*_1_, *x*_2_, *x*_3_, *γ*_1_, *γ*_2_, *γ*_3_, *ω*_1_, *ω*_2_, and *ω*_3_ are the amplitudes, damping rates and resonance frequencies of the resonance modes of M1, M2 and M3, respectively. κ_12_ and κ_23_ represent the coupling coefficients between the resonances of M1-M2 and M2-M3, respectively. g_1_, g_2_ and g_3_ are the geometric parameters indicating the strength each square ring couples to the incident field E. By solving Eq. (1), we obtain:


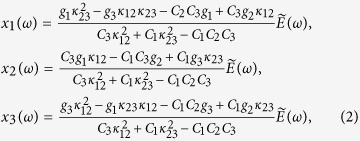


with


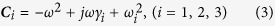


The electromagnetic polarizability of the samples is expressed as


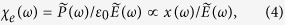


where *P*(*ω*) is the intensity of polarization, *ε*_0_ is the permittivity of vacuum. The total resonance amplitude *x*(*ω*) = *x*_*1*_(*ω*) + *x*_*2*_(*ω*) + *x*_*3*_(*ω*) is the linear superposition of the constituent resonators. By substituting Eqs. (1), (2) and (3) into Eq. (4), the polarizability 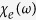 of the samples can be expressed using the resonance and mutual coupling coefficients of each CRR *γ*_*i*_, *ω*_*i*_, g_*i*_, (_*i*_ = 1,2,3) and κ_12_, κ_23_. The polarizability of the active metamaterial layer with a thickness of *d* is 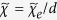.

In the measurements, the obtained amplitude transmission can be expressed as


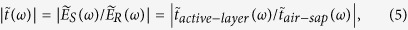


where 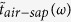 is the transmission at the air-sapphire interface of the reference sample, which can be directly obtained using the Fresnel coefficients:


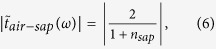


considering that the active layer is an effective medium with thickness *d*, around which are air and sapphire substrate, respectively. As the active layer is thin enough, the Fabry-Perot interference transmission equation can be used to express the transmission distribution:


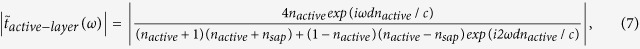


where 
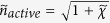
 and 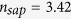 are the refractive indices of the active metamaterial layer and lossless sapphire substrate, respectively. *c* is the light velocity in vacuum.

Since *d* ~ 500 nm is much smaller than the wavelengths of the terahertz waves, the *d→*0 limit of the transmission coefficient can be adapted to evaluate the measured transmission, as presented below^12^:


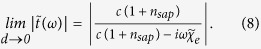


**Figure 6:**
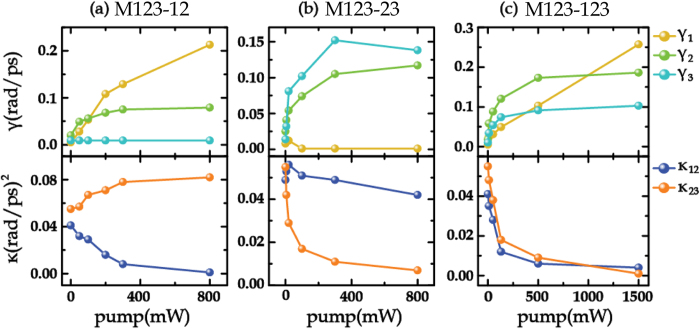
Theoretical fitting values of electromagnetic parameters γ_1_, γ_2_, γ_3_, κ_12_, and κ_23_ under different pump powers for (a) M123-12, (b) M123-23 and (c) M123-123.

The fitting results using Eq. (8), as shown in Figs. 3(b), 4(b) and 5(b), display a good agreement to the experimental measurements. As the coupling between M1 and M3 is very weak, only the coupling between two adjacent rings (i.e. M12, M23) was taken into account by the coupled Lorentz oscillator model in these proposed metamaterials. The fitting parameters with respect to different photoexcitation fluences are plotted in Fig. 6. It is observed that, for the sample M123-12 (see Fig. 6(a)), the damping rate γ_1_ shows a significant increase while γ_2_ grows up gently when bridging the outermost two rings with the photoexcited Si islands, which induces a decreasing coupling coefficient κ_12_. The damping rate γ_3_ does not change obviously with the increasing photoexcitation fluence. Additionally, the resonance of M3 enhances with increasing pump power, accompanied by the attenuation in resonances for M1 and M2. All these finally lead to a slightly enhanced coupling between M2 and M3, namely κ_23_ increases slightly with pump fluence. For the sample M123-23 with photoexcited Si islands bridging the innermost two rings (see Fig. 6(b)), γ_2_ and γ_3_ increase markedly and κ_23_ decreases greatly with respect to the pump power. Although a little enhancement in the resonance for M1 occurs, it would not enhance the coupling between M1 and M2, partly due to a larger ring spacing between M1 and M2 than that between M2 and M3. This is why κ_12_ slightly decreases in this case. For the sample M123-123, all the parameters γ_1_, γ_2_, γ_3_, κ_12_ and κ_23_ exhibit strong dependence on the increased photoexcitation fluence, as shown in Fig. 6(c). The damping rates of all the resonance modes of M1, M2 and M3 increase distinctly with pump power, which results in the decrease in both of κ_12_ and κ_23_. The variation trends of these parameters shown in Fig. 6 indicate that the active modulation arises from the enhanced loss of the resonances in the rings connected by the photoexcited Si islands and the reduced coupling between the two bridged rings. Therefore, it is concluded that, when the pump power is strong enough, the enhanced *γ*_*i*_ (*i* = *1,2,3*) not only suppresses self-mode excitation, but also leads to complete disappearance of the transmission windows by reducing the mode-coupling strength κ_*j*,*j + *1_ (*j* = 1,2).

In the simulations, the photoexcited Si islands were modeled with a pump-power-dependent conductivity. When the pump power was increased from 0 to 800 mW, the conductivity of the Si islands was varied from 25 to 2.2 × 10^4^ S m^−1^. The experimental results agree well with the numerical simulations, as shown in Figs. 3(c), 4(c), and 5(c).

## Conclusion

We have demonstrated the attractive dynamic mode coupling effect that offers an active and ultrafast way to control the near fields between the resonators, therefore enabling reconfigurable metamaterials with on-demand desirable optical properties. Such dynamic manipulation of the near field mode coupling schemes using an active material can open up fascinating opportunities for active imaging, plasmon rulers, lasing spacers, active sensors, filters, modulators and active polarization rotation devices.

This work was partially supported by Cooperative Innovation Center of Terahertz Science, the National Basic Research Program of China (Grant No. 2014CB339800), the National Natural Science Foundation of China (Grant Nos 61307125, 61107053, 61138001, 61427814, 61422509, 61420106006, and 61328503), the Major National Development Project of Scientific Instruments and Equipment (Grant No. 2011YQ150021), the U. S. National Science Foundation (Grant No. ECCS-1232081), the Program for Changjiang Scholars and Innovative Research Team in University, “PCSIRT” (Grant No. IRT13033), and the Guangxi Key Laboratory of Automatic Detecting Technology and Instruments (Grant No. YQ14207).

## Additional Information

**How to cite this article**: Su, X. *et al.* Dynamic mode coupling in terahertz metamaterials. *Sci. Rep.* **5**, 10823; doi: 10.1038/srep10823 (2015).

## References

[b1] Pendry J. B. Negative refraction makes a perfect lens. Phys. Rev. Let. 85, 3966 (2000). 11041972 10.1103/PhysRevLett.85.3966

[b2] Shelby R. A., Smith D. R. & Schultz S. Experimental verification of a negative index of refraction. Science 292, 5514 (2001). 10.1126/science.1058847 11292865

[b3] Zhang S. *et al.* Experimental demonstration of near-infrared negative-index metamaterials. Phys. Rev. Lett. 95, 137404 (2005). 16197179 10.1103/PhysRevLett.95.137404

[b4] Fang N., Lee H., Sun C. & Zhang X. Sub-diffractionlimited optical imaging with a silver superlens. Science 308, 5721 534–537 (2005). 15845849 10.1126/science.1108759

[b5] Pendry J. B., Schurig D. & Smith D. R. Controlling electromagnetic fields. Science 312, 5781 1780–1782 (2006). 16728597 10.1126/science.1125907

[b6] Schurig D. *et al.* Metamaterial electromagnetic cloak at microwave frequencies. Science 314, 5801 977–980 (2006). 17053110 10.1126/science.1133628

[b7] Ergin T., Stenger N., Brenner P., Pendry J. B. & Wegener M. Three-dimensional invisibility cloak at optical wavelengths. Science 328, 5976 337–339 (2010). 20299551 10.1126/science.1186351

[b8] Tao H. *et al.* A metamaterial absorber for the terahertz regime: design, fabrication and characterization. Opt. Express 16, 7181–7188 (2008). 18545422 10.1364/oe.16.007181

[b9] O’Hara J. F. *et al.* Thin-film sensing with planar terahertz metamaterials: sensitivity and limitations. Opt. Express 16, 1786–1795 (2008). 18542258 10.1364/oe.16.001786

[b10] Debus C. & Bolivar P. H. Frequency selective surfaces for high sensitivity terahertz sensing. Appl. Phys.Lett. 91, 184102 (2007).

[b11] Zhang S., Genov D. A., Wang Y., Liu M. & Zhang X. Plasmon-induced transparency in metamaterials. Phys. Rev. Lett. 101, 047401 (2008). 18764363 10.1103/PhysRevLett.101.047401

[b12] Zhu Z. H. *et al.* Broadband plasmon induced transparency in terahertz metamaterials. Nanotechnology 24, 214003 (2013). 23618809 10.1088/0957-4484/24/21/214003

[b13] Tassin P., Zhang L., Koschny T., Economou E. N. & Soukoulis C. M. Low-loss metamaterials based on classical electromagnetically induced transparency. Phys. Rev. Lett. 102, 053901 (2009). 19257513 10.1103/PhysRevLett.102.053901

[b14] Liu N. *et al.* Plasmonic analogue of electromagnetically induced transparency at the Drude damping limit. Nature Mater. 8, 758 –762 (2009). 19578334 10.1038/nmat2495

[b15] Liu X. J. *et al.* Electromagnetically induced transparency in terahertz plasmonic metamaterials via dual excitation pathways of the dark mode. Appl. Phys. Lett. 100, 131101 (2012).

[b16] Li Z. Y. *et al.* Manipulating the plasmon-induced transparency in terahertz metamaterials. Opt. Express 19, 8912–8919 (2011). 21643144 10.1364/OE.19.008912

[b17] Ma Y. F. *et al.* Plasmon-induced transparency in twisted Fano terahertz metamaterials. Opt. Mat. Express 1, 391–399 (2011). 10.1364/OE.19.008912 21643144

[b18] Yu N. F. *et al.* Light propagation with phase discontinuities: generalized laws of reflection and refraction. Science 334, 333 (2011). 21885733 10.1126/science.1210713

[b19] Aieta F. *et al.* Out-of-plane reflection and refraction of light by anisotropic optical antenna metasurfaces with phase discontinuities. Nano Lett. 12, 1702−1706 (2012). 22335616 10.1021/nl300204s

[b20] Zhang X. Q. *et al.* Broadband terahertz wave deflection based on C-shape complex metamaterials with phase discontinuities. Adv. Mater. 25, 4567 (2013). 23787976 10.1002/adma.201204850

[b21] Liu L. X. *et al.* Broadband metasurfaces with simultaneous control of phase and amplitude. Adv. Mater. 1, 1 (2014). 10.1002/adma.201401484 24863731

[b22] Rubén M. & Arjan W. K. Myth or Reality? fixation of carbon dioxide into complex organic matter under mild conditions. Chem. Int. Ed. 49, 9822–9838 (2011). 10.1002/cssc.201100102 21567978

[b23] Daniel M. C. & Astruc D. Gold nanoparticles: Assembly, supramolecular chemistry, quantum-size-related properties, and applications toward biology, catalysis, and nanotechnology. Chem. Rev. 104, 293–346 (2004). 14719978 10.1021/cr030698+

[b24] Singh R., Rockstuhl C., Lederer F. & Zhang W. L. The impact of nearest neighbor interaction on the resonances in terahertz metamaterials. Appl. Phys. Lett. 94, 021116 (2009).

[b25] Chiam S., Singh R., Zhang W. L. & Bettiol A. A. Controlling metamaterial resonances via dielectric and aspect ratio effects. Appl. Phys. Lett. 97, 191906 (2010).

[b26] Chowdhury D. R. *et al.* Tailored resonator coupling for modying the terahertz metamaterial response. Opt. Express 19, 10679–10685 (2011). 21643323 10.1364/OE.19.010679

[b27] Al-Naib I. *et al.* Conductive Coupling of Split Resonators: A path to THz metamaterials with ultrasharp resonances. Phys. Rev. Lett. 112, 183903 (2014). 24856698 10.1103/PhysRevLett.112.183903

[b28] Papasimakis N., Fu Y. H., Fedotov V. A., Prosvirnin S. L. & Tsai D. P. Metamaterial with polarization and direction insensitive resonant transmission response mimicking electromagnetically induced transparency. Appl. Phys. Lett. 94, 211902 (2009).

[b29] Kim J., Soref R. & Buchwald W. R. Multi-peak electromagnetically induced transparency (EIT)-like transmission from bull’s-eye-shaped metamaterial. Opt. Express 18, 17997–18002 (2010). 20721186 10.1364/OE.18.017997

